# Supplementary material for: Successful Practices in Performing and Maintaining Physical Activity in Adults with Type 2 Diabetes Mellitus: A Qualitative Study
Source: Int J Environ Res Public Health. 2022 Oct 28;19(21):14041. doi: 10.3390/ijerph192114041 (PMC9658001; doi:10.3390/ijerph192114041)
Supplement: Supplementary file 1 [file ijerph-19-14041-s001.zip › ijerph-1966226-supplementary.pdf]

Supplementary Material S1. Interview questions

I would like to talk about your experience as a person who suffers from DM2 and the physical activity (PA) you do for your care. But first, how is the treatment of DM2 composed of PA, medication and diet?
How do you think PA influences the DM2 process? What relationship do you think there is between DM2 and PA?
What does PA mean to you? What type of AF does it perform?
What changes did you have to make to maintain the realization of PA?
What social and environmental support did you have to perform PA?
What difficulties did you encounter when practicing PA?
What motivations did I have to start the PA?
What support did you have to realize PA?
How would you describe sedentary behavior?
How would you describe the role of the nurse in this process?
